# Genome-wide association study of myopia progression in Chinese adolescents and application of polygenic risk score prediction

**DOI:** 10.3389/fchem.2026.1778732

**Published:** 2026-04-21

**Authors:** Qinye Liu, Hui Shen, Xinglun Dang, Mike Zhongyu He, Yizhou Wei, Yingyun Shi, Xiaoyu Wei, Fen Chen, Di Han, Chengyong Liu, Jia Hu, Weina Liu

**Affiliations:** 1 Department of Nutrition and Food Hygiene, School of Public Health, Southeast University, Nanjing, China; 2 Suzhou Center for Disease Control and Prevention, Suzhou, China; 3 Department of Psychosomatics and Psychiatry, Zhongda Hospital, School of Medicine, Advanced Institute for Life and Health, Southeast University, Nanjing, China; 4 Department of Environmental Medicine, Icahn School of Medicine at Mount Sinai, New York, NY, United States; 5 School of Biomedical Engineering and Informatics, Nanjing Medical University, Nanjing, China; 6 Affiliated Hospital of Nanjing University of Chinese Medicine, Liyang Branch of Jiangsu Province Hospital of Chinese Medicine, Nanjing, China

**Keywords:** asian screening array chip, Chinese adolescents, genome-wide association study, myopia progression, polygenic risk score

## Abstract

In recent years, the prevalence of myopia has sharply increased in East Asia, emerging as a major public health issue. This study aimed to identify genetic risk factors for myopia progression and to develop polygenic risk score (PRS) models to predict myopia progression risk. Genotyping was performed using the Asian Screening Array chip among 294 Chinese adolescents who completed a 2-year follow-up. A two-stage (discovery cohort: N = 176; replication cohort: N = 118) genome-wide association study (GWAS) was subsequently conducted. Functional annotation and MAGMA analysis were performed to confirm biological relevance of the identified loci in the progression of myopia. Based on GWAS results from the discovery cohort and a European population from the United Kingdom Biobank (N = 460,536), we constructed single-ancestry and cross-ancestry PRS models with PRSice-2 and PRS-CSx. We evaluated the predictive performance of these models using the replication cohort. Our meta-analysis identified seven novel suggestive loci associated with myopia progression, including *FSTL5* on 4q32.2, *SMARCA2* on 9p24.3, *CCDC3* on 10p13, *GALNT6/ACVR1B* on 12q13.13, *CRY1* on 12q23.3, *ULK2* on 17p11.2, and *MYL4/EFCAB13-DT* on 17q21.32. For myopia progression risk prediction in East Asians, PRS analysis showed that the East Asian training dataset (*R* ^2^: 5.69%; OR: 1.61, 95% CI: 1.06–2.44; AUC: 0.66) outperformed both the European and cross-ancestry datasets. This study identified seven promising loci associated with myopia progression and demonstrated that PRS exhibited enhanced predictive performance in genetically and phenotypically matched populations. Our findings expand the genetic understanding of myopia progression in East Asian adolescents and provide new insights for myopia prevention and control.

## 1 Introduction

Myopia is a refractive error that primarily occurs during childhood and adolescence. It is characterized by excessive elongation of the axial length of the eyeball, in which parallel light rays converge anterior to the retina, leading to visual blur (Flitcroft et al., 2019). Myopia is a leading ocular disorder globally, with the highest prevalence observed in children and adolescents in Asia. It is estimated that approximately 80%–90% of school-aged children in Asia suffer from myopia, with 10%–20% affected by high myopia (Baird et al., 2020). It is predicted that the prevalence of myopia will continue to rise, reaching 50% globally and 10% for high myopia by 2050 (Holden et al., 2016). Progression to high myopia markedly increases the risk of macular degeneration, retinal detachment, glaucoma, and even blindness (Ohno-Matsui et al., 2021), while also imposing a substantial economic burden on society due to the costs associated with optical correction and clinical management (Ma et al., 2022). It is essential to control myopia progression early in life.

The etiology of myopia is multifactorial, involving both genetic predispositions and environmental influences (Baird et al., 2020). Early identification of high-risk children and adolescents with genetic susceptibility is essential to optimize preventive and therapeutic strategies (Han et al., 2022). To date, the NHGRI-EBI GWAS Catalog (https://www.ebi.ac.uk/gwas/) (Sollis et al., 2023) has reported approximately 250 loci associated with myopia and related traits that reach genome-wide significance (*P* < 5.0 × 10^−8^) in genome-wide association studies (GWAS). However, most genetic variants remain unidentified. In 2020, a GWAS meta-analysis involving 542,934 individuals from European populations identified 336 new genetic markers related to refractive errors, with rs12193446 on the *LAMA2* gene showing the strongest association (Hysi et al., 2020). Li et al. (2022) genotyped 1,347 myopic patients and 453 controls in Guangxi to investigate *MTOR* and *PDGFRA*, identifying rs1057079 and rs1064261 (*MTOR*) as variants associated with mild to moderate myopia. A recent East Asian GWAS identified *LILRB2* on 19q13.42 as a novel locus implicated in pathological myopia (Jiang L. et al., 2024). Overall, studies on myopia-related genetic polymorphisms in Asian populations are predominantly focused on candidate-gene analyses, with markedly fewer GWAS conducted in Asian-ancestry cohorts compared to European counterparts, and evidence on myopia progression in children and adolescents remains limited. Furthermore, polygenic risk scores (PRS) leveraging ancestry-matched or cross-ancestry myopia GWAS summary statistics have demonstrated utility in predicting myopia-related traits (Chen et al., 2021; Kassam et al., 2022; Lanca et al., 2021; Lin et al., 2024). However, their performance for predicting myopia progression in Asian populations remains to be validated. In this study, we conducted a two-stage GWAS analysis on myopia progression in Chinese adolescents and constructed PRS models across different populations to elucidate the genetic architecture of myopia progression.

## 2 Materials and methods

### 2.1 Participants

A total of 294 first-year students were recruited from five secondary schools in Suzhou, with baseline data collected in September 2020 and the final follow-up conducted in March 2023. All participants underwent annual refraction assessments by professional ophthalmologists using an autorefractor (RM-800 [TOPCON, Tokyo, Japan]). Participants demonstrating ≥1.0 D myopic progression over 2 years were classified as cases, whereas those with <1.0 D progression were designated as controls (Chen et al., 2021; Saxena et al., 2023; Verkicharla et al., 2023). Participants had no history of congenital eye diseases, family history of glaucoma, ocular trauma, organic lesions such as corneal or retinal conditions, or previous eye treatments or surgeries. This study employed a two-stage design, comprising a discovery stage and a replication stage. The discovery cohort included 89 cases and 87 controls from three secondary schools in Suzhou. The replication cohort consisted of 59 cases and 59 controls from the remaining two secondary schools. Cases and controls were frequency-matched based on sex and baseline age (±1 year).

The study procedures adhered to the principles of the Declaration of Helsinki and were approved by the Ethics Committee of the Suzhou Center for Disease Control and Prevention (Ethics approval number SZJK2022-XW001). All participants were fully informed about the study content and provided written informed consent.

### 2.2 Genotyping and quality control

Genomic DNA was extracted from human peripheral blood samples according to standard laboratory protocols. Genotyping of the 294 individuals in both the discovery and replication stages was performed using the Infinium Asian Screening Array-24 v1.0 chip (Illumina, San Diego, California, United States). This chip, developed by Illumina, is optimized for the genomic characteristics of East Asian populations and contains approximately 660,000 fixed marker sites. Quality control (QC) was conducted using PLINK 1.9 (Purcell et al., 2007). Samples with discrepant sex information, call rates <97%, and heterozygosity deviating by more than 3 standard deviations from the mean were excluded. The cryptic relationships between samples were estimated based on identity by descent, and the sample with the lower call rate in any pair with a pi-hat >0.1875 was removed. In addition, principal component analysis (PCA) was conducted using the smartpca program from EIGENSOFT v7.2.1 (https://hsph.harvard.edu/research/price-lab/software/) (Patterson et al., 2006; Price et al., 2006). Firstly, we merged genotype data from myopia progression cases and controls with the CHB/CHS data from the 1,000 Genomes Phase 3 (Abecasis et al., 2012) for ancestral PCA analysis (Supplementary Figure S1). Subsequently, we conducted PCA for the myopia progression cases and controls, excluding the MHC region (chr6: 25 MB–34 MB, hg19) (Supplementary Figure S2). The first ten PCs were calculated for each iteration, and five iterations were performed. Samples with one or more PCs deviating by more than 6 standard deviations from the mean were excluded. The impact of different PCs on the GWAS summary statistics was evaluated through the genomic inflation factor (λ_GC_), and the first seven PCs were selected as covariates.

Variant QC was performed based on the following criteria: call rate <97%, significant differences in missing data between cases and controls (*P* < 1.0 × 10^−5^), significant deviation from Hardy-Weinberg equilibrium in the control group (*P* < 1.0 × 10^−5^), and a minor allele frequency <0.01 were excluded. After rigorous quality control, a total of 465,936 autosomal variants (89 cases and 87 controls) were included for subsequent imputation analysis.

### 2.3 Genotype imputation

Phasing was performed using SHAPEIT2 (Delaneau et al., 2011). Genotype imputation was performed using Minimac3 (Das et al., 2016), with the 1,000 Genomes Phase 3 version 5 reference panel. After imputation, variants with an imputation quality score <0.7 and those with a minor allele frequency <0.01 were excluded. A total of 6,569,222 autosomal variants were included in the subsequent association analysis.

### 2.4 Association analysis

Genetic association analysis for the discovery stage was performed using an additive logistic regression model in PLINK 1.9, adjusting for sex, baseline age, and the first seven PCs as covariates. Quantile-quantile plots and Manhattan plots were generated using the CMplot package in the R statistical software, version 4.4.1 (https://github.com/YinLiLin/CMplot).

### 2.5 Replication study and meta-analysis

A replication study was conducted in an independent sample, using the same QC standards as in the discovery stage. After stringent QC, 458,439 variants from 59 cases and 59 controls were retained and subsequently imputed. Variants from the discovery GWAS with *P* < 1.0 × 10^−4^ (Supplementary Table S1) were included in the replication analysis. Meta-analysis of the two stages was performed using the inverse-variance weighted fixed-effects model in METAL (version 2011-03-25) (https://genome.sph.umich.edu/wiki/METAL) (Willer et al., 2010). Regional association plots for significant variants were generated using the LocusZoom website (http://locuszoom.org/) (Pruim et al., 2010). Power was calculated using the CaTS Power Calculator (https://csg.sph.umich.edu//abecasis/CaTS/index.html) (Skol et al., 2006) with the sample sizes in a two-stage design (discovery: N = 176; replication: N = 118). Under an additive genetic model and a suggestive significance threshold (*P* = 1.0 × 10^−4^), the odds ratio (OR) required to achieve 80% power in the two-stage analysis ranged from 2.00 to 3.67 for variants with minor allele frequencies of 0.10, 0.20, 0.30, and 0.40.

### 2.6 Linkage disequilibrium score regression analysis

In GWAS, linkage disequilibrium score regression (LDSC) analysis aims to differentiate the effects of polygenic factors from confounding factors (Bulik-Sullivan et al., 2015). The reference LD scores for East Asians were downloaded from the open digital repository (https://zenodo.org/records/10515792) and the analysis was performed on the discovery GWAS.

### 2.7 Functional annotation

Functional annotation of significant variants was performed using HaploReg v4.2 (https://pubs.broadinstitute.org/mammals/haploreg/haploreg.php) (Ward and Kellis, 2016) and RegulomeDB 2.2 (https://www.regulomedb.org/regulome-search/) (Boyle et al., 2012). On 16 March 2025, the associations between the identified variants and gene expression regulation were investigated using expression quantitative trait locus and splice quantitative trait locus data from the GTEx Portal v10 online database (https://www.gtexportal.org/home/) (GTEx Consortium, 2013).

### 2.8 MAGMA analysis

To examine the enrichment of myopia progression GWAS associations in specific functions or pathways, we performed gene set analysis on the discovery GWAS using MAGMA v1.1 (https://cncr.nl/research/magma/) (de Leeuw et al., 2015). Gene Ontology and Kyoto Encyclopedia of Genes and Genomes (GO/KEGG) entries were downloaded from the MSigDB v2024.1 database (https://www.gsea-msigdb.org/gsea/index.jsp) (Liberzon et al., 2015).

### 2.9 Polygenic risk score analysis

Two GWAS results were used as the base data. The first dataset was the myopia progression GWAS from 176 East Asian participants in the discovery stage (denoted as EAS). The second dataset was the myopia GWAS (GWAS ID: ukb-b-6353) from 460,536 European samples (denoted as EUR), obtained from OpenGWAS (https://gwas.mrcieu.ac.uk/). Genotype data from 118 East Asian samples in the replication stage were used as the target data. The PRS was constructed using the clumping and thresholding method in PRSice-2 (Choi and O'Reilly, 2019). The clumping parameters were set as follows: -clump-kb 250, --clump-p 1, --clump-r2 0.1. The variance explained by the model was quantified using Nagelkerke *R* ^2^ and Liability *R* ^2^ (Lee et al., 2012), with the myopia prevalence in Chinese high school students (81.2%) used as the population prevalence parameter (Xinhua News Agency, 2024). Logistic regression was used to estimate the OR and 95% confidence intervals (CI) for each standard deviation increase in PRS, and the area under the receiver operating characteristic curve (AUC) was computed. Covariates included sex, baseline age, and the first seven PCs.

Currently, large-scale GWAS summary statistics predominantly focus on European ancestry, and cross-ancestry genetic heterogeneity often limits the predictive performance of PRS in non-European ancestry groups (Duncan et al., 2019). To address this, we applied PRS-CSx (Ruan et al., 2022), combining GWAS from European and East Asian populations (denoted as EUR + EAS), and generated posterior effect estimates for each single nucleotide polymorphism using a Bayesian regression model. We then used PLINK 2 --score command to compute polygenic scores for 118 East Asian individuals and assessed the predictive accuracy of the model.

## 3 Results

### 3.1 General demographic characteristics

A total of 148 cases and 146 controls from two stages passed QC. No significant differences were observed between the groups in sex, baseline age, and baseline refractive status (*P* > 0.05). The average baseline age of the case group was 15.64 ± 0.34 years, with 47.97% male and 52.03% female participants. The mean baseline age of the control group was 15.66 ± 0.34 years, including 48.63% males and 51.37% females. The baseline refractive status and refractive progression during the follow-up period are shown in Table 1.

**TABLE 1:** Demographic and ocular characteristics of participants.

Dataset	Total		Discovery stage		Replication stage	
	Cases	Controls	Cases	Controls	Cases	Controls
Number of samples	148	146	89	87	59	59
Sex						
Male	71 (47.97%)	71 (48.63%)	43 (48.31%)	43 (49.43%)	28 (47.46%)	28 (47.46%)
Female	77 (52.03%)	75 (51.37%)	46 (51.69%)	44 (50.57%)	31 (52.54%)	31 (52.54%)
Baseline age	15.64 ± 0.34	15.66 ± 0.34	15.59 ± 0.33	15.59 ± 0.30	15.71 ± 0.36	15.77 ± 0.36
Spherical equivalent (diopters)						
OD^a^ (2020)	−3.55 ± 2.10	−3.33 ± 2.16	−3.42 ± 2.05	−3.11 ± 2.02	−3.66 ± 2.15	−3.79 ± 2.38
OS^a^ (2020)	−3.12 ± 2.25	−2.98 ± 2.28	−3.07 ± 2.14	−2.68 ± 2.14	−3.16 ± 2.37	−3.58 ± 2.46
OD^a^ (2023)	−4.66 ± 2.01	−3.34 ± 2.27	−4.40 ± 2.00	−3.21 ± 2.13	−4.89 ± 2.00	−3.61 ± 2.53
OS^a^ (2023)	−4.36 ± 2.32	−3.05 ± 2.25	−4.09 ± 2.20	−2.80 ± 2.11	−4.60 ± 2.42	−3.54 ± 2.47
Myopia progression (diopters)						
OD^a^	1.11 ± 0.92	0.01 ± 0.91	0.97 ± 0.66	0.10 ± 0.58	1.23 ± 1.10	−0.18 ± 1.34
OS^a^	1.24 ± 0.72	0.07 ± 0.74	1.02 ± 0.40	0.12 ± 0.51	1.44 ± 0.88	−0.04 ± 1.05

^a^ OD: right eye; OS: left eye.

### 3.2 Genome-wide association study and replication analysis

**FIGURE 1:**
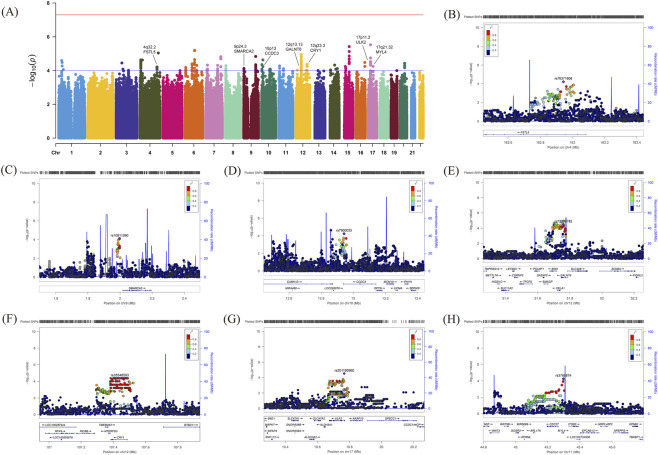
GWAS-identified regions in the discovery stage. **(A)** Manhattan plot of myopia progression GWAS in the discovery phase. The X-axis represents the chromosomes on which the genetic variants are located, and the Y-axis shows the -log_10_(*P*) values for all variants. The blue horizontal line represents the suggestive *P*-threshold (*P* = 1.0 × 10^−4^), while the red horizontal line represents the genome-wide significant *P*-threshold (*P* = 5.0 × 10^−8^). **(B–H)** LocusZoom plots of the myopia progression-related genetic regions. The lead single nucleotide polymorphisms and insertion-deletion variants are represented as purple diamonds. Variant colors indicate LD with lead variants: red (*r* ^2^ ≥ 0.8), orange (0.6 ≤ *r* ^2^ < 0.8), green (0.4 ≤ *r* ^2^ < 0.6), light blue (0.2 ≤ *r* ^2^ < 0.4), dark blue (*r* ^2^ < 0.2), and gray (unknown).

In the discovery stage, 6,569,222 autosomal variants from 89 progression cases and 87 controls underwent QC and imputation for downstream association testing. The λ_GC_ was 1.063, indicating minimal inflation of the test statistics (Supplementary Figure S3). LDSC yielded an intercept of 1.003, which was very close to 1, indicating that inflation was driven by polygenicity with negligible population stratification (Bulik-Sullivan et al., 2015). We detected 430 variants with suggestive association (*P* < 1.0 × 10^−4^; Figure 1A) and advanced them to replication.

To validate the discovery stage results, we conducted a replication study on 430 variants (*P* < 1.0 × 10^−4^) in an independent sample of myopia progression (59 cases and 59 controls). Meta-analysis of the two stages revealed seven novel loci with suggestive significant associations with myopia progression (Table 2), including *FSTL5* on 4q32.2 (lead variant: rs76371606, *P* = 1.32 × 10^−5^); *SMARCA2* on 9p24.3 (lead variant: rs10811260, *P* = 2.93 × 10^−5^); *CCDC3* on 10p13 (lead variant: rs7900033, *P* = 4.32 × 10^−6^); *GALNT6* on 12q13.13 (lead variant: rs73309782 and the variant in the same LD block: rs58448629, *P* = 1.32 × 10^−5^); *CRY1* on 12q23.3 (lead variant: rs35548593, *P* = 5.09 × 10^−5^); *ULK2* on 17p11.2 (lead variant: rs201190960, *P* = 1.74 × 10^−6^); and *MYL4* on 17q21.32 (lead variant: rs3785874, *P* = 7.34 × 10^−5^) (Figures 1B–H). Additionally, we provide a list of all variants with suggestive associations (*P* < 1.0 × 10^−4^) for myopia progression from both stages in Supplementary Table S2.

**TABLE 2:** Association results of the 7 loci identified in this study.

Variant	Chr^a^: Pos^a^ (GRCh37)	Gene (location)	Region	A1/A2	Discovery stage (89 cases/87 controls)			Replication stage (59 cases/59 controls)			Meta-analysis (148 cases/146 controls)		
					AF^a^	*P*	OR^a^ (95% CI)	AF	*P*	OR (95% CI)	*P*	OR	I^b^
rs76371606	4:162944170	*FSTL5* (4q32.2)	Intron	C/A	0.26/0.10	6.13 × 10^−5^	3.99 (2.03–7.85)	0.19/0.10	0.04	2.21 (1.04–4.68)	1.32 × 10^−5^	3.06	24.15
rs10811260	9:1996894	*SMARCA2* (9p24.3)	Intergenic	A/G	0.42/0.21	8.17 × 10^−5^	2.83 (1.69–4.74)	0.38/0.30	0.07	1.76 (0.95–3.25)	2.93 × 10^−5^	2.32	25.29
rs7900033	10:12942295	*CCDC3* (10p13)	Intron	A/G	0.43/0.22	5.82 × 10^−5^	2.85 (1.71–4.75)	0.36/0.24	0.02	2.20 (1.13–4.30)	4.32 × 10^−6^	2.59	0
rs73309782	12:51759858	*GALNT6* (12q13.13)	Intron	C/T	0.24/0.07	3.48 × 10^−5^	4.54 (2.22–9.30)	0.17/0.08	0.07	2.27 (0.94–5.49)	1.32 × 10^−5^	3.45	30.19
rs58448629	12:51760686	*GALNT6* (12q13.13)	Intron	C/T	0.24/0.07	3.48 × 10^−5^	4.54 (2.22–9.30)	0.17/0.08	0.07	2.27 (0.94–5.49)	1.32 × 10^−5^	3.45	30.19
rs35548593	12:107439903	*CRY1* (12q23.3)	Intron	TA/T	0.37/0.17	4.33 × 10^−5^	3.48 (1.92–6.34)	0.36/0.23	0.10	1.65 (0.91–2.98)	5.09 × 10^−5^	2.39	67.04
rs201190960	17:19767785	*ULK2* (17p11.2)	Intron	C/CA	0.22/0.44	2.98 × 10^−5^	0.34 (0.21–0.56)	0.29/0.43	0.01	0.46 (0.25–0.86)	1.74 × 10^−6^	0.38	0
rs3785874	17:45297113	*MYL4* (17q21.32)	Intron	T/G	0.37/0.59	5.61 × 10^−5^	0.38 (0.24–0.61)	0.42/0.52	0.17	0.68 (0.39–1.18)	7.34 × 10^−5^	0.49	58.66

^a^ Abbreviations: Chr, chromosome; Pos, position; AF, allele frequency (case AF/control AF); OR, odds ratio.

^b^ I2 heterogeneity index of meta-analysis.

### 3.3 Functional evaluation

The rs10811260 is located in an intergenic region, whereas the remaining seven variants are located within introns. These variants in non-coding regions may significantly impact gene expression. The HaploReg v4.2 database predicted that one variant was located in a promoter histone mark region, four variants were located in enhancer histone mark regions, one was located in a DNase hypersensitive site, and all eight variants were predicted to alter transcription factor binding motifs (Table 3), suggesting their potential regulatory functions. Additionally, the RegulomeDB 2.2 database assigned strong regulatory evidence to rs3785874 (*MYL4*), rs76371606 (*FSTL5*), and rs35548593 (*CRY1*), with ranking scores of 3a, 4, and 5 and model scores of 0.60, 0.61, and 0.62, respectively. These findings indicated that the highlighted variants may modulate transcription factor binding; by contrast, the remaining five variants lacked significant evidence of binding (Table 3). The GTEx Portal v10 database revealed that rs73309782 and rs58448629 modulate *ACVR1B* expression downstream of *GALNT6*, while rs3785874 affects expression of *MYL4* and its downstream gene *EFCAB13-DT* (Table 3).

**TABLE 3:** Functional annotation of the 8 variants identified in this study.

Variant	Chr: Pos (GRCh37)	Gene (location)	Region	HaploReg				RegulomeDB		GTEx portal						
				Promoter histone marks	Enhancer histone marks	DNAse	Motifs changed	Ranking score^e^	Model score^f^	eQTL^a^			sQTL^a^			
										*P*	Gene symbol	Tissue	*P*	Gene symbol	Tissue	Intron ID
rs76371606	4:162944170	*FSTL5* (4q32.2)	Intron	​	​	​	AIRE, Nkx2	4	0.61	​	​	​	​	​	​	​
rs10811260	9:1996894	*SMARCA2* (9p24.3)	Intergenic	​	​	​	SRF	6	0.00	​	​	​	​	​	​	​
rs7900033	10:12942295	*CCDC3* (10p13)	Intron	​	BRN^a^	​	Hoxa7	5	0.13	​	​	​	​	​	​	​
rs73309782	12:51759858	*GALNT6* (12q13.13)	Intron	​	BLD^a^	​	4 altered motifs^c^	7	0.18	3.1 × 10^−5^	*ACVR1B*	Brain - cortex	​	​	​	​
rs58448629	12:51760686	*GALNT6* (12q13.13)	Intron	​	BLD, THYM^a^	​	Smad3, TAL1	6	0.20	3.1 × 10^−5^	*ACVR1B*	Brain - cortex	​	​	​	​
rs35548593	12:107439903	*CRY1* (12q23.3)	Intron	​	​	​	5 altered motifs^d^	5	0.62	​	​	​	​	​	​	​
rs201190960	17:19767785	*ULK2* (17p11.2)	Intron	​	​	​	HDAC2	6	0.30	​	​	​	​	​	​	​
rs3785874	17:45297113	*MYL4* (17q21.32)	Intron	BLD, GI^a^	8 tissues^a,b^	HRT, BLD^a^	LBP-1	3a	0.60	1.3 × 10^−5^	*EFCAB13-DT*	Adipose - visceral (omentum)	4.1 × 10^−12^	*MYL4*	Testis	47219748:47219904: clu_52416_+

^a^ Abbreviations: ADRL, adrenal gland; BLD, blood; BRN, brain; ESDR, embryonic stem cells derived; GI, gastrointestinal tract; HRT, heart; MUS, muscle; SPLN, spleen; THYM, thymus; eQTL, expression quantitative trait locus; sQTL, splice quantitative trait locus.

^b^ BLD, ESDR, ADRL, HRT, GI, MUS, THYM, SPLN.

^c^ Pax-4, Pax-5, TCF12, Zec.

^d^ Foxp1, HDAC2, Hoxa4, Zfp105, p300.

^e^ Ranking score: 3a, TF, binding + any motif + chromatin accessibility peak; 4, TF, binding + chromatin accessibility peak; 5, TF, binding or chromatin accessibility peak; 6, Motif hit; 7, Other.

^f^ Model score: range from 0 to 1, with 1 being most likely to be a regulatory variant.

### 3.4 GO/KEGG gene enrichment analysis

The discovery GWAS enriched several GO and KEGG terms, including reticulophagy (*P* = 3.41 × 10^−5^), negative regulation of protein maturation (*P* = 9.49 × 10^−5^), regulation of retrograde transport, endosome to Golgi (*P* = 2.13 × 10^−4^), peptidase inhibitor complex (*P* = 9.25 × 10^−4^), eukaryotic translation initiation factor 3 complex (EIF3M) (*P* = 1.06 × 10^−3^), regulation of autophagy (*P* = 1.15 × 10^−3^), and other GO/KEGG terms (Supplementary Table S3), suggesting that the risk variants may promote myopia progression by regulating protein metabolism and autophagic apoptosis.

### 3.5 Polygenic risk score prediction evaluation

**FIGURE 2:**
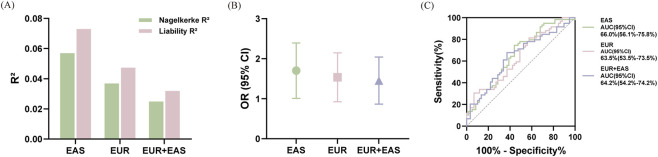
Accuracy of genetic risk prediction using different baseline datasets. **(A)** Each bar represents a test dataset, with the green bars indicating Nagelkerke *R* ^2^ and the pink bars indicating Liability *R* ^2^ (with a population myopia risk of 0.812). **(B)** The OR for each standard deviation increase in PRS across different test datasets, with error bars indicating the 95% CI. Green circles represent the EAS test set, pink squares represent the EUR test set, and purple triangles represent the EUR + EAS test set. **(C)** Receiver operating characteristic curves for different training sets (green = EAS, pink = EUR, purple = EUR + EAS).

We compared three PRS models and selected the optimal results. When using the EAS training set, the *P*-value threshold (*P*_T_) was 0.001, and the genetic variance (estimated by Nagelkerke *R* ^2^) was 5.69%. For the EUR training set, the genetic variance was 3.69% (*P*_T_ = 5.0 × 10^−8^). Using the cross-ancestry EUR + EAS training set, the genetic variance was 2.49% (global shrinkage parameter, weight = auto) (Figure 2A). Based on the prevalence of myopia (81.2%) among Chinese adolescents, liability-scale *R* ^2^ values were 7.30%, 4.73%, and 3.19% for the EAS, EUR, and EUR + EAS models, respectively (Figure 2A). Furthermore, the EAS GWAS (OR: 1.61, 95% CI: 1.06–2.44, *P* = 0.026; AUC: 0.66) demonstrated greater effect size and stronger discriminatory power compared to the EUR (OR: 1.46, 95% CI: 0.97–2.18, *P* = 0.069; AUC: 0.64) and EUR + EAS GWAS (OR: 1.37, 95% CI: 0.91–2.08, *P* = 0.131; AUC: 0.64) (Figure 2B,C). Overall, the PRS derived from the EAS GWAS performed the best.

## 4 Discussion

We conducted a GWAS on myopia progression in Chinese adolescents and identified seven novel loci suggestively associated with myopia progression: *FSTL5* (4q32.2), *SMARCA2* (9p24.3), *CCDC3* (10p13), *GALNT6/ACVR1B* (12q13.13), *CRY1* (12q23.3), *ULK2* (17p11.2), and *MYL4/EFCAB13-DT* (17q21.32). Additionally, the PRS models indicated that the EAS training set outperformed both the EUR and the cross-ancestry EUR + EAS training sets in predicting myopia progression risk of East Asian populations.

This study identified *SMARCA2* (lead variant: rs10811260) as associated with myopia progression. Previous studies have demonstrated that mutations in *SMARCA2* on chromosome 9p24.3 can result in an autosomal dominant genetic disorder, Nicolaides-Baraitser syndrome (Gao et al., 2019; Pan et al., 2021; Van Houdt et al., 2012). Ocular features of Nicolaides-Baraitser syndrome mainly include myopia, hypermetropia, and periorbital skin ptosis (Simmers et al., 2022). *SMARCA2* is the catalytic subunit of the SWI/SNF chromatin remodeling complex, which plays a crucial role in regulating embryonic development (Gao et al., 2019). Studies in mice and zebrafish have demonstrated that the SWI/SNF complex is closely involved in the development of the brain, eyes, and heart (Bögershausen and Wollnik, 2018). Notably, a previous study identified a copy number variation (≈350 kb) in the 9p24.3 chromosomal region associated with pathological myopia (Zhou et al., 2012), suggesting that *SMARCA2*, located in the same region, may be a pathogenic gene involved in myopia development. Furthermore, the HaploReg database predicted that rs10811260 may alter the regulatory motif bound by *SRF*. *SRF* is a key regulator of the transcription of α-smooth muscle actin and collagen genes (Miano et al., 2007). It was shown that increased *SRF* activity in guinea pig scleral fibroblasts induced myofibroblast transdifferentiation and upregulated α-smooth muscle actin expression (Yuan et al., 2018), thereby promoting myopic scleral remodeling. These findings suggest that rs10811260-*SMARCA2* may be a true risk locus for myopia progression.

The myopia progression-related variants, rs73309782 and rs58448629, in the *GALNT6/ACVR1B* region on 12q13.13, significantly affected the regulatory expression of *ACVR1B* (GTEx Portal v10, *P* = 3.1 × 10^−5^). Additionally, the HaploReg database indicated that rs58448629 may alter the binding motif of *Smad3*. *ACVR1B* is a member of the *TGF-β* type I receptor family and primarily mediates the activin A signaling pathway (Hillege et al., 2022). Activated *ACVR1B* has been shown to regulate downstream *SMAD* signaling in the fibrosis of organs such as muscle (Ly et al., 2020), skin (Ramachandran et al., 2021), and bone (Jobling et al., 2009), promoting the differentiation of fibroblasts into myofibroblasts. It is widely accepted that *TGF-β* binds to receptor proteins on the surface of scleral fibroblasts, promoting their transdifferentiation into myofibroblasts, which is a key pathological mechanism underlying scleral remodeling (McBrien, 2013). Therefore, rs73309782 and rs58448629 may regulate *ACVR1B* expression and its downstream *SMAD* signaling, acting synergistically with the *TGF-β* pathway to contribute to scleral matrix remodeling and the development of myopia.

We identified rs35548593 at the *CRY1* locus (12q23.3) as associated with myopia progression. *CRY1* encodes a core circadian clock protein essential for maintaining circadian rhythm (Felder-Schmittbuhl et al., 2018; Griffin et al., 1999). Emerging evidence suggests that circadian rhythms are linked to eye development and myopia (Chakraborty et al., 2018; Nilsen et al., 2022; Troilo et al., 2019). Thompson et al. (2003) used real-time RT-PCR to demonstrate *CRY1* mRNA expression in the human macula and peripheral retina. Wong et al. (2018) performed immunohistochemistry on mouse retinal sections and detected widespread *CRY1* expression in cone cells, long photoreceptor cells, and some ganglion cells, across all retinal layers. Recently, Bartölke et al. (2025) confirmed the presence of *CRY1* in the retinas of both humans and chimpanzees through immunohistochemistry. Furthermore, when chicks were fitted with a −10.0 D lens in one eye to induce myopia, *CRY1* expression in the retina of the myopic eye remained largely unchanged within 24 h compared to the control eye, losing its typical circadian rhythm expression (Stone et al., 2020). In *CRY1* and *CRY2* double-knockout mice, the absence of circadian rhythms resulted in reduced sensitivity to pupillary light response (Owens et al., 2012). Thus, rs35548593-*CRY1* may be involved in the pathogenesis of myopia progression through the regulation of circadian rhythms.

Among the other newly identified loci, *FSTL5* on 4q32.2 belongs to the *FSTL* family and is an extracellular matrix-secreted protein. *FSTL* family proteins are involved in regulating cell migration, proliferation, and differentiation (Chen et al., 2025). Additionally, the molecular structure of *FSTL5* is similar to that of *FST*. It has been shown in liver cells that *FST* binds to activin A from the *TGF-β* family and inhibits downstream *SMAD* signaling, thereby exerting anti-fibrotic effects and reducing the differentiation of hepatic fibroblasts into myofibroblasts (Katz et al., 2016; Kreidl et al., 2009; Patella et al., 2006; Zhang et al., 2020). It could be hypothesized that *FSTL5* (lead variant: rs76371606) may play a similar role in scleral fibroblasts, potentially offering protection against myopia progression by modulating fibrotic responses. *CCDC3* on 10p13 is a protein secreted by adipose tissue and the vascular system, playing multiple roles in lipid metabolism, abdominal obesity, and fatty liver (Omari et al., 2023). Notably, *CCDC3* mRNA was highly expressed in obese mice and in those fed a high-fat, high-sucrose diet (Kobayashi et al., 2010). Increased *CCDC3* mRNA expression was also been observed in the visceral adipose tissue of abdominally obese men (Ugi et al., 2014). Meanwhile, numerous epidemiologic studies have demonstrated that obesity is a major risk factor for the development of myopia (Harrington et al., 2019; Peled et al., 2022; Yin et al., 2025). Therefore, rs7900033-*CCDC3* may influence myopia progression through the regulation of lipid metabolism. *ULK2* on 17p11.2 is involved in autophagy initiation, with its transcript expressed in tissues such as the brain, eyes, and heart (Dong et al., 2024). Recent studies have demonstrated links between autophagy and ocular diseases (Jaggi et al., 2022; Jiang B. et al., 2024; Zhang et al., 2025), suggesting that rs201190960-*ULK2* may play a critical role in myopia progression by influencing autophagic processes within ocular cells. *MYL4* on 17q21.32 encodes myosin, a key component of muscle fibers, with its expression in extraocular muscles persisting from development into adulthood (Schiaffino et al., 2015). Currently, *MYL4* primarily functions in contraction, movement, and morphological maintenance in the atria and skeletal muscles (Ciecierska et al., 2020; Zhong et al., 2023). Many ocular tissues, including the ciliary muscle, also contain myosin. Other studies indicated that both humans with myopia from prolonged near work and animal models of induced myopia exhibited a contraction-thinning phenomenon in the ciliary muscle (Pucker et al., 2020; Wagner et al., 2019). *EFCAB13-DT* encodes a long non-coding RNA, the function of which remains unclear. Nevertheless, long non-coding RNAs can influence gene expression through various mechanisms, such as transcriptional regulation, chromatin remodeling, and post-translational modifications of proteins (Herman et al., 2022), playing a role in the pathogenesis of various eye diseases (Wu et al., 2024). These functional roles suggest that rs3785874 in the *MYL4/EFCAB13-DT* region may influence myopia progression through the regulation of muscle function and gene expression in ocular tissues. However, the precise mechanisms by which these loci contribute to the progression of myopia require further investigation.

Our PRS analysis demonstrated that, for predicting myopia progression risk in East Asians, the EAS model (*R* ^2^: 5.69%; OR: 1.61, 95% CI: 1.06–2.44; AUC: 0.66) outperformed both the EUR (*R* ^2^: 3.69%; OR: 1.46, 95% CI: 0.97–2.18; AUC: 0.64) and the cross-ancestry EUR + EAS training sets (*R* ^2^: 2.49%; OR: 1.37, 95% CI: 0.91–2.08; AUC: 0.64). The myopia PRS, based on a GWAS training set of 10,156 individuals and a validation set of 6,170 individuals from Taiwan, revealed AUC values of 0.80, 0.78 and 0.73 for high myopia, moderate myopia, and mild myopia, respectively, with each unit increase in PRS corresponding to an 11% increased risk of high myopia (OR: 1.11, 95% CI: 1.03–1.20) (Lin et al., 2024). Ghorbani Mojarrad et al. (2020) constructed a myopia PRS model using a GWAS training set of 287,448 individuals and a validation set of 1,516 individuals from the United Kingdom, which indicated AUC of 0.67 and *R* ^2^ of 7.1%. Our EAS model exhibited slightly inferior predictive performance, likely due to the smaller sample size, which reduced statistical power. Larger sample sizes will be required to further investigate the predictive performance of myopia progression PRS. Additionally, a myopia PRS based on European myopia GWAS for Singaporean Chinese children showed AUC of 0.77 for high myopia and 0.62 for moderate myopia (Lanca et al., 2021). Chen et al. (2021) utilized three Asian myopia loci to construct a myopia progression PRS for Hong Kong children, which explained 2.2% of *R* ^2^. Individuals with PRS in the sixth quantile had 2.26 times higher odds of myopia progression as compared with those in the first quantile (OR: 2.26, 95% CI: 1.50–3.42). Asian high myopia and myopic macular degeneration PRS models based on European myopia GWAS demonstrated AUC of 0.73 and 0.66, respectively (Kassam et al., 2022). The predictive accuracy of PRS models constructed across different ancestries or phenotypes was comparatively modest, likely attributable to genetic heterogeneity between populations and phenotypes. Empirical and theoretical studies have indicated that an increase in genetic distance between the training and validation samples is associated with a decrease in predictive performance (Scutari et al., 2016; Wang et al., 2020). In this study, the genetic heterogeneity between European and East Asian populations is considerable, and since myopia and myopia progression may have distinct genetic bases, these factors might play a crucial role in diminishing the predictive power of PRS. However, other studies have shown that the PRS-CSx algorithm can substantially improve the accuracy of cross-ancestry PRS models (Ge et al., 2022; Ruan et al., 2022). In contrast, our results indicated that the PRS-CSx model performed less effectively, potentially because we employed the “auto” and “meta” version of the PRS-CSx algorithm, which can reduce predictive performance but still offer utility in cases with limited sample sizes in the target dataset (Ruan et al., 2022). Notably, due to the high prevalence of myopia among Chinese adolescents, the genetic variance explained increased when we converted Nagelkerke *R* ^2^ to Liability *R* ^2^ (Figure 2A). Overall, myopia progression PRS studies remain relatively limited, whereas PRS research for myopia and high myopia has generally improved with the expansion of large-scale GWAS training datasets. However, prediction accuracy remains highly dependent on ancestry and phenotype definitions, and transferability across populations or related phenotypes is often attenuated. Therefore, current efforts increasingly emphasize larger ancestry-matched GWAS and more effective cross-ancestry modeling methods to improve generalizability and predictive accuracy.

Our study is the first GWAS on myopia progression in Asian adolescents, filling a significant gap in current research and offering new insights for myopia prevention and control. However, this study also has several limitations. First, the definition of myopia progression in this study was relatively simplistic, focusing solely on changes in refractive error from baseline to 2 years of follow-up. In practice, true myopia progression also involves axial-length elongation and dynamic refractive-change rates. Future studies would benefit from collecting axial length data to more precisely characterize progression, distinguish structural elongation from refractive fluctuation, and support refined phenotype definitions, including axial elongation rate. Additionally, using alternative dioptric-change cutoffs or modeling dioptric change as a continuous outcome would alter the case-control classification and power, which could affect the estimated effect sizes and statistical significance of the associations, indicating the need for further analyses using alternative phenotype definitions. Second, since behavioral exposures highly relevant to myopia progression, such as outdoor time and near work, were not included in the association model, some residual confounding and gene-environment interaction may remain, which could affect the magnitude and robustness of the association signals. Third, our sample size was modest relative to other myopia GWAS, and our power analysis indicated that this study was primarily powered to detect variants with relatively large effects at the suggestive threshold, potentially missing smaller-effect associations. Fourth, the loci we identified reached only suggestive significance and did not meet the genome-wide threshold. Nevertheless, it is widely acknowledged that the biological findings from GWAS are not solely dependent on the strength of statistical associations, as demonstrated in prior studies (Liu et al., 2023; Schneider et al., 2017). Where GWAS findings were validated through functional gene experiments. Additionally, the PRS developed in our study showed significant associations. Consequently, our findings provide preliminary evidence that these seven loci may be involved in myopia progression, warranting further investigation in larger cohorts and functional studies. Fifth, we included the lead single nucleotide polymorphisms from three myopia GWAS in Supplementary Table S4. However, none of these variants reached statistical significance in our discovery GWAS. Sixth, this study conducted a preliminary exploration of East Asian myopia progression risk prediction using East Asian myopia progression GWAS and European myopia GWAS. Although the EAS model outperformed the EUR and EUR + EAS models, its discrimination was modest (AUC = 0.66), limiting clinical utility and suitability for real-world risk stratification as a stand-alone tool. Future large-scale GWAS of Asian myopia progression, along with integrated models incorporating non-genetic predictors, are needed to validate and extend these findings. Finally, as all participants were recruited from Suzhou, our findings should be generalized with caution to other East Asian and non-East Asian populations.

## 5 Conclusion

This study identified seven novel loci suggestively associated with myopia progression and demonstrated that the predictive performance of PRS was superior in genetically and phenotypically homogeneous populations. These findings enhance the understanding of genetic factors underlying myopia progression in East Asian adolescents. Future research should replicate these results in larger and more diverse populations and undertake functional validation to pinpoint targets for preventing myopia onset and progression.

## Data Availability

The original contributions presented in the study are publicly available. This data can be found here: http://ftp.ebi.ac.uk/pub/databases/gwas/summary_statistics/GCST90726001-GCST90727000/GCST90726612.
