# Supplementary material for: Genome-wide association study of myopia progression in Chinese adolescents and application of polygenic risk score prediction
Source: Front Chem. 2026 Apr 21;14:1778732. doi: 10.3389/fchem.2026.1778732 (PMC13139945; doi:10.3389/fchem.2026.1778732)
Supplement: Supplementary file 2 [file DataSheet1.docx]

Supplementary Material

1. **Supplementary Figures and Tables**
   1. **Supplementary Figures**


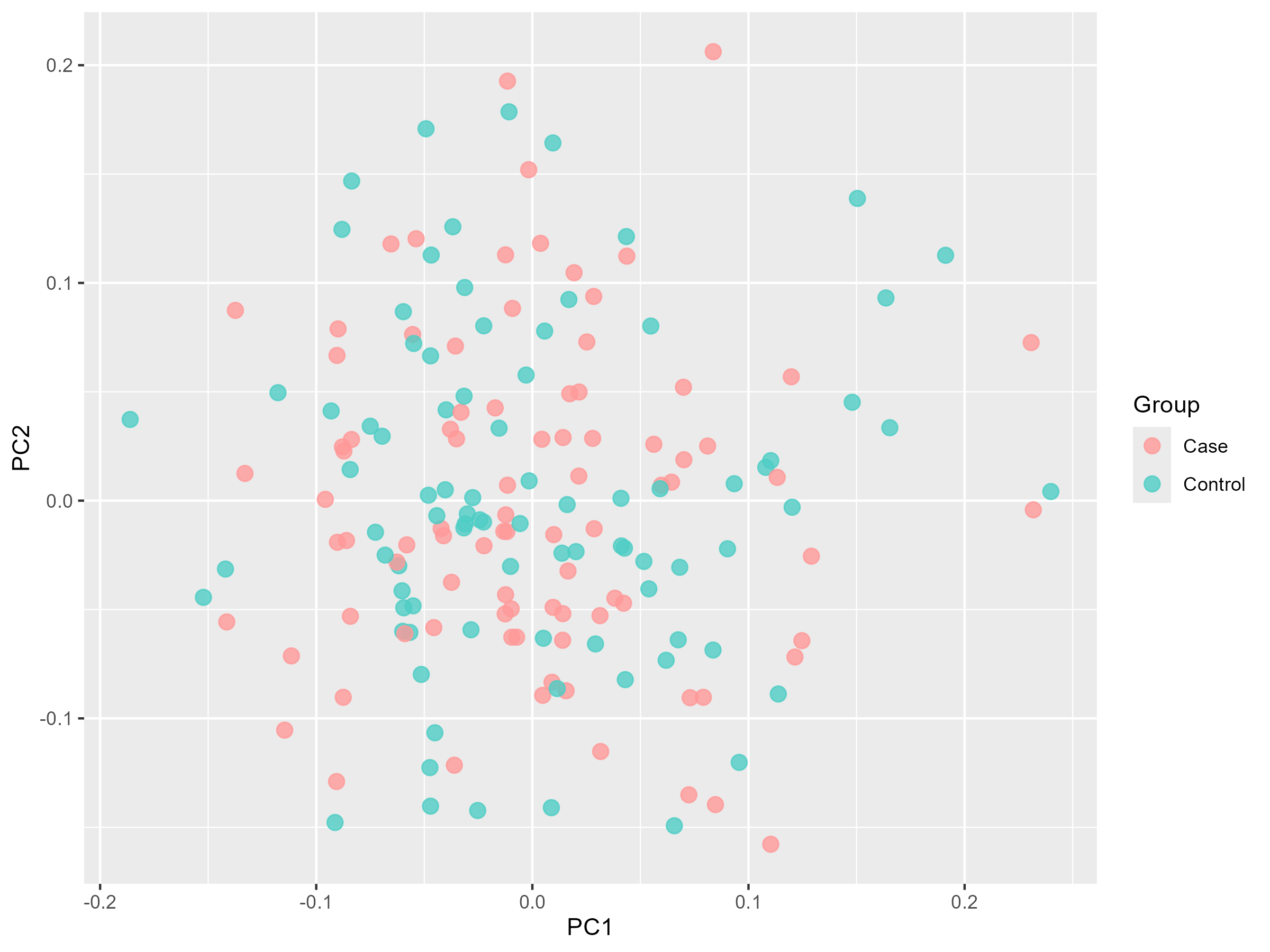


**Supplementary Figure S1.** PCA plot for the discovery stage samples of this study and the 1000 Genomes Project samples. The X-axis represents the PC1, and the Y-axis represents the PC2. Green circles represent the Chinese population (CHB/CHS) from the 1000 Genomes Project Phase 3, red circles represent myopia progression cases from the discovery cohort, and blue circles represent healthy controls from the same cohort. The three sample groups clustered together, indicating that the discovery stage samples had Chinese Han ancestry.


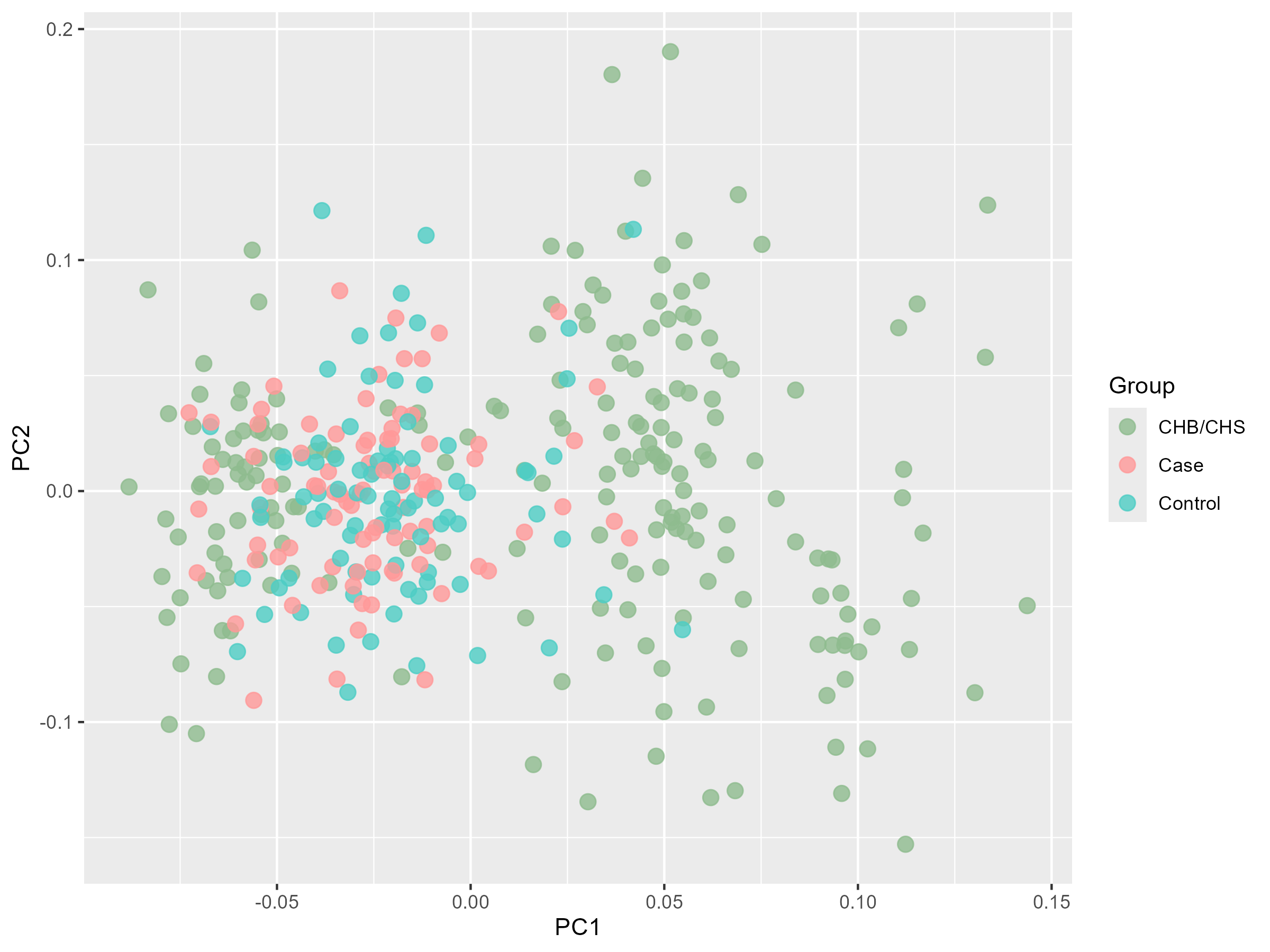


**Supplementary Figure S2.** PCA plot of myopia progression cases and healthy controls from the discovery stage. Red and blue circles represent the myopia progression cases and healthy controls from the discovery cohort, respectively. The cases and controls formed a tight cluster, suggesting no population stratification in the discovery samples.


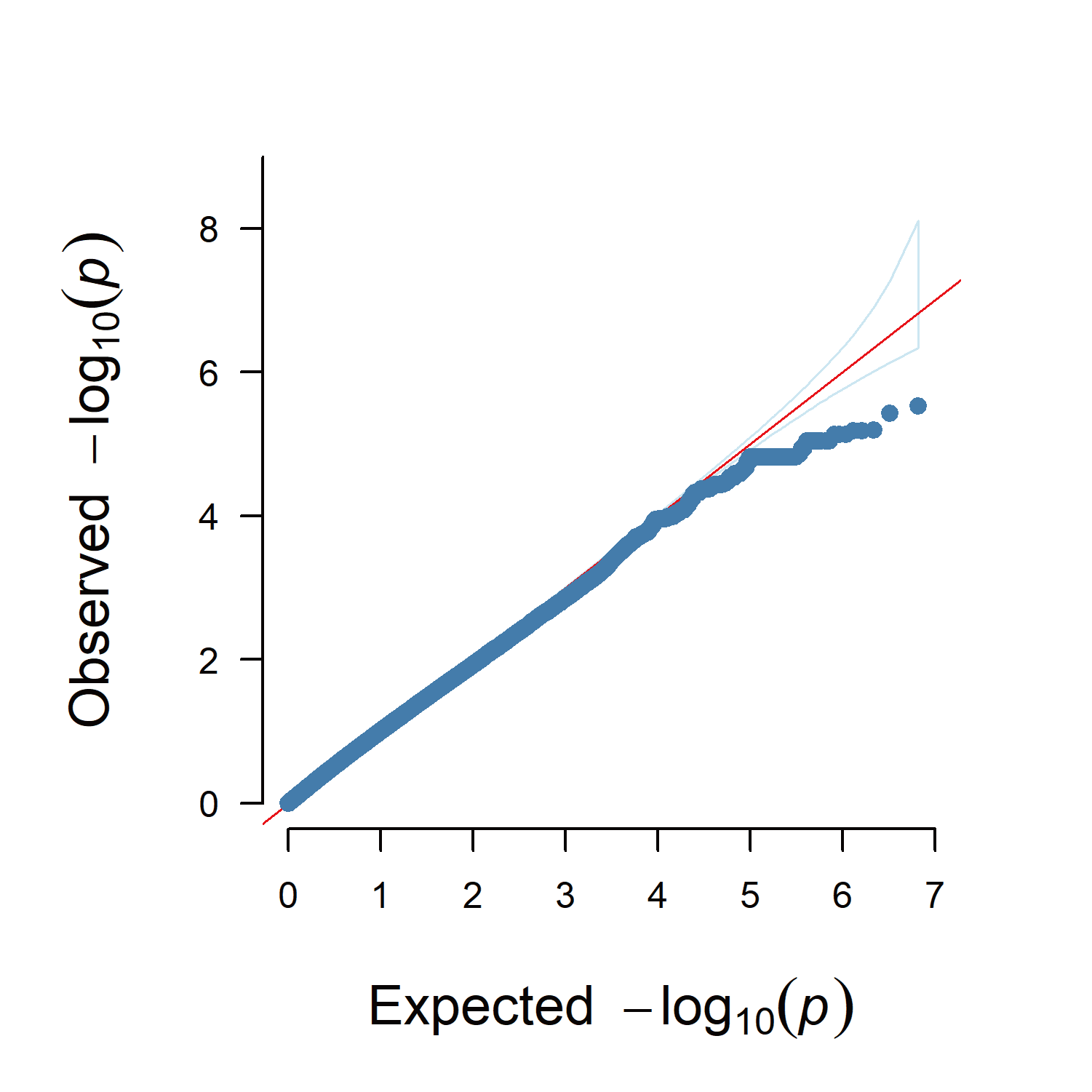


**Supplementary Figure S3.** Quantile-quantile plot of the discovery GWAS. The X-axis represents the expected -log_10_(*P*) under the null hypothesis, and the Y-axis represents the observed -log_10_(*P*). The red line represents X = Y. The light blue area indicates the 95% CI.

- 1. **Supplementary Tables**

**Supplementary Table S2.** Variants suggestively associated with myopia progression (*P* < 1.0 × 10^-4^) in the two-stage analysis (discovery and replication).

|  |  |  |  |  | **Discovery stage**  **(89 cases/87 controls)** | | | **Replication stage**  **(59 cases/59 controls)** | | | **Meta-analysis**  **(148 cases/146 controls)** | | |
| --- | --- | --- | --- | --- | --- | --- | --- | --- | --- | --- | --- | --- | --- |
| **Variant** | **Chr**^a^**: Pos**^a^  **(GRCh37)** | **Gene**  **(Location)** | **Region** | **A1/A2** | **AF**^a^ | ***P*** | **OR**^a^**(95% CI)** | **AF** | ***P*** | **OR(95% CI)** | ***P*** | **OR** | **I**^b^ |
| rs76371606 | 4:162944170 | *FSTL5*  (4q32.2) | intron | C/A | 0.26/0.10 | 6.13×10^-5^ | 3.99(2.03–7.85) | 0.19/0.10 | 0.04 | 2.21(1.04–4.68) | 1.32×10^-5^ | 3.06 | 24.15 |
| rs72372775 | 4:162954959 | *FSTL5*  (4q32.2) | intron | AT/A | 0.26/0.10 | 7.06×10^-5^ | 4.01(2.02–7.95) | 0.17/0.10 | 0.08 | 2.00(0.93–4.30) | 3.40×10^-5^ | 2.94 | 43.36 |
| rs76511338 | 4:162955379 | *FSTL5*  (4q32.2) | intron | A/T | 0.26/0.10 | 8.61×10^-5^ | 3.84(1.96–7.52) | 0.17/0.11 | 0.10 | 1.87(0.88–3.96) | 6.06×10^-5^ | 2.79 | 49.08 |
| rs56400540 | 9:1996560 | *SMARCA2*  (9p24.3) | regulatory region | C/G | 0.40/0.20 | 7.46×10^-5^ | 2.86(1.70–4.81) | 0.36/0.29 | 0.14 | 1.58(0.86–2.89) | 7.28×10^-5^ | 2.22 | 53.23 |
| rs10811260 | 9:1996894 | *SMARCA2*  (9p24.3) | intergenic | A/G | 0.42/0.21 | 8.17×10^-5^ | 2.83(1.69–4.74) | 0.38/0.30 | 0.07 | 1.76(0.95–3.25) | 2.93×10^-5^ | 2.32 | 25.29 |
| rs7900033 | 10:12942295 | *CCDC3*  (10p13) | intron | A/G | 0.43/0.22 | 5.82×10^-5^ | 2.85(1.71–4.75) | 0.36/0.24 | 0.02 | 2.20(1.13–4.30) | 4.32×10^-6^ | 2.59 | 0 |
| rs7903718 | 10:12942508 | *CCDC3*  (10p13) | intron | T/C | 0.43/0.22 | 5.82×10^-5^ | 2.85(1.71–4.75) | 0.36/0.24 | 0.02 | 2.20(1.13–4.30) | 4.32×10^-6^ | 2.59 | 0 |
| rs149802233 | 12:51692340 | *BIN2*  (12q13.13) | intron | A/T | 0.25/0.09 | 7.74×10^-5^ | 3.84(1.97–7.48) | 0.19/0.11 | 0.13 | 1.88(0.83–4.25) | 5.95×10^-5^ | 2.88 | 43.46 |
| rs75409038 | 12:51747005 | *GALNT6*  (12q13.13) | 3'-UTR | T/C | 0.24/0.07 | 6.21×10^-5^ | 4.11(2.06–8.19) | 0.15/0.09 | 0.20 | 1.78(0.74–4.30) | 8.05×10^-5^ | 2.99 | 53.18 |
| rs60044798 | 12:51747465 | *GALNT6*  (12q13.13) | 3'-UTR | C/T | 0.24/0.07 | 6.21×10^-5^ | 4.11(2.06–8.19) | 0.15/0.09 | 0.20 | 1.78(0.74–4.30) | 8.05×10^-5^ | 2.99 | 53.18 |
| rs12582216 | 12:51747914 | *GALNT6*  (12q13.13) | 3'-UTR | A/G | 0.24/0.07 | 6.21×10^-5^ | 4.11(2.06–8.19) | 0.15/0.09 | 0.20 | 1.78(0.74–4.30) | 8.05×10^-5^ | 2.99 | 53.18 |
| rs3741700 | 12:51752231 | *GALNT6*  (12q13.13) | intron | T/C | 0.23/0.07 | 7.47×10^-5^ | 4.12(2.05–8.31) | 0.15/0.09 | 0.20 | 1.78(0.74–4.30) | 9.70×10^-5^ | 2.99 | 53.13 |
| rs142817819 | 12:51756888 | *GALNT6*  (12q13.13) | intron | A/AC | 0.25/0.07 | 2.71×10^-5^ | 4.47(2.22–8.99) | 0.16/0.09 | 0.14 | 1.97(0.81–4.76) | 2.45×10^-5^ | 3.26 | 50.88 |
| rs73309782 | 12:51759858 | *GALNT6*  (12q13.13) | intron | C/T | 0.24/0.07 | 3.48×10^-5^ | 4.54(2.22–9.30) | 0.17/0.08 | 0.07 | 2.27(0.94–5.49) | 1.32×10^-5^ | 3.45 | 30.19 |
| rs73309783 | 12:51760103 | *GALNT6*  (12q13.13) | intron | A/G | 0.23/0.07 | 5.82×10^-5^ | 4.30(2.11–8.75) | 0.16/0.08 | 0.11 | 2.07(0.86–5.01) | 3.42×10^-5^ | 3.23 | 37.29 |
| rs73309787 | 12:51760183 | *GALNT6*  (12q13.13) | intron | G/A | 0.24/0.07 | 3.48×10^-5^ | 4.54(2.22–9.30) | 0.17/0.08 | 0.07 | 2.27(0.94–5.49) | 1.32×10^-5^ | 3.45 | 30.19 |
| rs73309789 | 12:51760208 | *GALNT6*  (12q13.13) | intron | G/A | 0.24/0.07 | 3.48×10^-5^ | 4.54(2.22–9.30) | 0.17/0.08 | 0.07 | 2.27(0.94–5.49) | 1.32×10^-5^ | 3.45 | 30.19 |
| rs73309790 | 12:51760264 | *GALNT6*  (12q13.13) | intron | A/G | 0.24/0.07 | 3.48×10^-5^ | 4.54(2.22–9.30) | 0.17/0.08 | 0.07 | 2.27(0.94–5.49) | 1.32×10^-5^ | 3.45 | 30.19 |
| rs2067764 | 12:51760436 | *GALNT6*  (12q13.13) | intron | G/A | 0.24/0.07 | 3.48×10^-5^ | 4.54(2.22–9.30) | 0.17/0.08 | 0.07 | 2.27(0.94–5.49) | 1.32×10^-5^ | 3.45 | 30.19 |
| rs61612083 | 12:51760530 | *GALNT6*  (12q13.13) | intron | A/G | 0.24/0.07 | 3.48×10^-5^ | 4.54(2.22–9.30) | 0.17/0.08 | 0.07 | 2.27(0.94–5.49) | 1.32×10^-5^ | 3.45 | 30.19 |
| rs58448629 | 12:51760686 | *GALNT6*  (12q13.13) | intron | C/T | 0.24/0.07 | 3.48×10^-5^ | 4.54(2.22–9.30) | 0.17/0.08 | 0.07 | 2.27(0.94–5.49) | 1.32×10^-5^ | 3.45 | 30.19 |
| rs73309797 | 12:51761925 | *GALNT6*  (12q13.13) | intron | T/A | 0.23/0.07 | 4.20×10^-5^ | 4.57(2.21–9.45) | 0.17/0.08 | 0.07 | 2.27(0.94–5.49) | 1.58×10^-5^ | 3.44 | 30.59 |
| rs12579841 | 12:51762370 | *GALNT6*  (12q13.13) | intron | G/T | 0.23/0.07 | 4.20×10^-5^ | 4.57(2.21–9.45) | 0.17/0.08 | 0.07 | 2.27(0.94–5.49) | 1.58×10^-5^ | 3.44 | 30.59 |
| rs12578858 | 12:51762834 | *GALNT6*  (12q13.13) | intron | T/C | 0.23/0.07 | 4.20×10^-5^ | 4.57(2.21–9.45) | 0.17/0.08 | 0.07 | 2.27(0.94–5.49) | 1.58×10^-5^ | 3.44 | 30.59 |
| rs35548593 | 12:107439903 | *CRY1*  (12q23.3) | intron | TA/T | 0.37/0.17 | 4.33×10^-5^ | 3.48(1.92–6.34) | 0.36/0.23 | 0.10 | 1.65(0.91–2.98) | 5.09×10^-5^ | 2.39 | 67.04 |
| rs201190960 | 17:19767785 | *ULK2*  (17p11.2) | intron | C/CA | 0.22/0.44 | 2.98×10^-5^ | 0.34(0.21–0.56) | 0.29/0.43 | 0.01 | 0.46(0.25–0.86) | 1.74×10^-6^ | 0.38 | 0 |
| rs3785874 | 17:45297113 | *MYL4*  (17q21.32) | intron | T/G | 0.37/0.59 | 5.61×10^-5^ | 0.38(0.24–0.61) | 0.42/0.52 | 0.17 | 0.68(0.39–1.18) | 7.34×10^-5^ | 0.49 | 58.66 |

^a^Abbreviations: Chr, chromosome; Pos, position; OR, odds ratio; AF: allele frequency (case AF/control AF). ^b^I^2^ heterogeneity index of meta-analysis.

**Supplementary Table S3.** GO/KEGG enrichment analysis of myopia progression in the discovery GWAS (top 13 and top 1 are shown).

| **FULL_NAME** | ***P*** |
| --- | --- |
| GOBP_RETICULOPHAGY | 3.41E-05 |
| GOBP_NEGATIVE_REGULATION_OF_PROTEIN_MATURATION | 9.49E-05 |
| GOBP_NEGATIVE_REGULATION_OF_INTERLEUKIN_17_PRODUCTION | 1.55E-04 |
| GOBP_REGULATION_OF_RETROGRADE_TRANSPORT_ENDOSOME_TO_GOLGI | 2.13E-04 |
| GOBP_POSITIVE_REGULATION_OF_INTRINSIC_APOPTOTIC_SIGNALING_PATHWAY | 2.33E-04 |
| GOBP_POSITIVE_REGULATION_OF_NATURAL_KILLER_CELL_ACTIVATION | 5.68E-04 |
| GOBP_N_ACYLPHOSPHATIDYLETHANOLAMINE_METABOLIC_PROCESS | 5.70E-04 |
| GOBP_CELLULAR_RESPONSE_TO_UV_C | 6.33E-04 |
| GOCC_CYTOPLASMIC_MICROTUBULE | 7.76E-04 |
| GOCC_PEPTIDASE_INHIBITOR_COMPLEX | 9.25E-04 |
| GOBP_EXOCRINE_PANCREAS_DEVELOPMENT | 9.37E-04 |
| GOBP_PULMONARY_VALVE_DEVELOPMENT | 1.06E-03 |
| GOCC_EUKARYOTIC_TRANSLATION_INITIATION_FACTOR_3_COMPLEX_EIF3M | 1.06E-03 |
| KEGG_REGULATION_OF_AUTOPHAGY | 1.15E-03 |

**Supplementary Table S4.** Association of genome-wide significant variants reported in previous myopia GWAS (East Asian or including East Asian samples) in the discovery stage of our study.

| **Study** | **Trait** | **Variant** | **Chr**^a^**: Pos**^a^  **(GRCh37)** | **A1/A2** | **AF**^a^ | ***P*** | **OR**^a^**(95% CI)** |
| --- | --- | --- | --- | --- | --- | --- | --- |
| Verma et al., 2024 | Myopia | rs310160 | 1:61322088 | A/T | 0.30/0.28 | 0.78 | 1.07(0.68–1.68) |
|  |  | rs2790110 | 1:200311674 | C/A | 0.42/0.49 | 0.13 | 0.68(0.42–1.12) |
|  |  | rs7545125 | 1:207424727 | G/A | 0.45/0.39 | 0.22 | 1.34(0.84–2.11) |
|  |  | rs12028838 | 1:219778675 | G/T | 0.39/0.36 | 0.56 | 1.15(0.73–1.81) |
|  |  | rs17713568 | 2:242132 | A/G | 0.25/0.22 | 0.11 | 6.13(0.67–55.69) |
|  |  | rs181661155 | 2:56089108 | G/A | Variant not available | | |
|  |  | rs6704590 | 2:172878554 | G/C | 0.07/0.08 | 0.60 | 0.80(0.35–1.85) |
|  |  | rs1550094 | 2:233385396 | G/A | 0.08/0.08 | 0.79 | 0.90(0.41–1.98) |
|  |  | rs6785073 | 3:141139330 | G/A | Variant not available | | |
|  |  | rs1139638 | 4:80827799 | A/C | Variant not available | | |
|  |  | rs2055178 | 4:81946533 | A/G | 0.26/0.19 | 0.18 | 1.41(0.85–2.33) |
|  |  | rs1034071 | 6:22097375 | C/T | 0.12/0.09 | 0.43 | 1.32(0.66–2.66) |
|  |  | rs7744813 | 6:73643289 | C/A | 0.15/0.18 | 0.29 | 0.72(0.39–1.33) |
|  |  | rs12193446 | 6:129820038 | A/G | Variant not available | | |
|  |  | rs16890057 | 8:40726582 | A/G | 0.10/0.10 | 0.96 | 1.02(0.48–2.16) |
|  |  | rs2582636 | 8:53376286 | A/G | 0.08/0.10 | 0.48 | 0.76(0.36–1.62) |
|  |  | rs10089517 | 8:60178721 | A/C | 0.48/0.51 | 0.54 | 0.88(0.58–1.33) |
|  |  | rs1340044 | 9:18362105 | T/A | Variant not available | | |
|  |  | rs7042950 | 9:77149837 | A/G | 0.21/0.27 | 0.17 | 0.68(0.40–1.18) |
|  |  | rs11594240 | 10:60284785 | G/C | Variant not available | | |
|  |  | rs11002137 | 10:79124594 | C/T | 0.17/0.17 | 0.74 | 0.90(0.48–1.69) |
|  |  | rs7903146 | 10:114758349 | T/C | 0.02/0.04 | 0.49 | 0.62(0.16–2.40) |
|  |  | rs11200630 | 10:124209684 | C/T | 0.41/0.44 | 0.47 | 0.84(0.52–1.35) |
|  |  | rs11602008 | 11:40149305 | T/A | 0.22/0.30 | 0.05 | 0.59(0.34–1.01) |
|  |  | rs11513245 | 12:9296354 | C/A | 0.06/0.08 | 0.47 | 0.74(0.32–1.70) |
|  |  | rs3138142 | 12:56115585 | T/C | 0.12/0.15 | 0.30 | 0.71(0.38–1.35) |
|  |  | rs724154 | 13:100673616 | A/G | 0.27/0.32 | 0.71 | 0.83(0.31–2.21) |
|  |  | rs66913363 | 14:54413001 | C/G | 0.24/0.28 | 0.38 | 0.79(0.47–1.33) |
|  |  | rs634990 | 15:35006073 | C/T | 0.52/0.44 | 0.14 | 1.39(0.90–2.16) |
|  |  | rs17648524 | 16:7459683 | C/G | 0.04/0.05 | 0.51 | 0.70(0.24–2.03) |
|  |  | rs2908972 | 17:11407259 | T/A | 0.42/0.45 | 0.49 | 0.85(0.54–1.33) |
|  |  | rs8075811 | 17:54735307 | A/G | 0.37/0.33 | 0.48 | 1.18(0.75–1.85) |
|  |  | rs12458939 | 18:42889835 | A/G | 0.30/0.24 | 0.23 | 1.33(0.84–2.11) |
|  |  | rs4808203 | 19:19568659 | C/T | 0.22/0.31 | 0.09 | 0.65(0.39–1.08) |
|  |  | rs9723267 | 22:46365557 | G/T | 0.28/0.34 | 0.37 | 0.79(0.47–1.32) |
| Zhao et al., 2021 | High myopia | rs10889602 | 1:66573381 | G/T | 0.06/0.03 | 0.24 | 1.96(0.63–6.05) |
|  |  | rs2193015 | 12:97563086 | T/C | 0.46/0.45 | 0.79 | 1.06(0.69–1.64) |
|  |  | rs9676191 | 18:49625503 | C/A | 0.13/0.12 | 0.81 | 1.09(0.55–2.14) |
| Meguro et al., 2020 | High myopia | rs698047 | 1:42333603 | G/C | 0.46/0.49 | 0.36 | 0.82(0.54–1.25) |
|  |  | rs2246661 | 1:204943991 | C/T | 0.26/0.28 | 0.99 | 0.10(0.61–1.62) |
|  |  | rs12032649 | 1:219778959 | T/G | 0.39/0.37 | 0.66 | 1.11(0.70–1.75) |
|  |  | rs17029206 | 3:1781516 | C/T | 0.37/0.36 | 0.94 | 0.98(0.61–1.57) |
|  |  | rs74633073 | 3:69278898 | T/C | 0.11/0.08 | 0.34 | 1.45(0.68–3.12) |
|  |  | rs76903431 | 12:130525867 | A/T | 0.06/0.05 | 0.65 | 1.25(0.48–3.27) |
|  |  | rs589135 | 15:35001442 | G/A | 0.50/0.43 | 0.20 | 1.32(0.87–2.01) |
|  |  | rs28415942 | 15:79384850 | C/T | 0.52/0.43 | 0.09 | 1.48(0.94–2.33) |
|  |  | rs72748160 | 15:86235882 | T/G | 0.04/0.03 | 0.60 | 1.39(0.41–4.79) |

^a^Abbreviations: Chr, chromosome; Pos, position; OR, odds ratio; AF: allele frequency (case AF/control AF).

**References**

Meguro, A., Yamane, T., Takeuchi, M., Miyake, M., Fan, Q., Zhao, W., et al. (2020). Genome-Wide Association Study in Asians Identifies Novel Loci for High Myopia and Highlights a Nervous System Role in Its Pathogenesis. *Ophthalmology* 127(12)**,** 1612-1624. doi: 10.1016/j.ophtha.2020.05.014.

Verma, A., Huffman, J.E., Rodriguez, A., Conery, M., Liu, M., Ho, Y.L., et al. (2024). Diversity and scale: Genetic architecture of 2068 traits in the VA Million Veteran Program. *Science* 385(6706)**,** eadj1182. doi: 10.1126/science.adj1182.

Zhao, F., Chen, W., Zhou, H., Reinach, P.S., Wang, Y., Juo, S.H., et al. (2021). PDE4B Proposed as a High Myopia Susceptibility Gene in Chinese Population. *Front. Genet.* 12**,** 775797. doi: 10.3389/fgene.2021.775797.
